# Connecting past to present: Examining different approaches to linking historical redlining to present day health inequities

**DOI:** 10.1371/journal.pone.0267606

**Published:** 2022-05-19

**Authors:** Clemens Noelke, Michael Outrich, Mikyung Baek, Jason Reece, Theresa L. Osypuk, Nancy McArdle, Robert W. Ressler, Dolores Acevedo-Garcia

**Affiliations:** 1 Heller School for Social Policy and Management, Brandeis University, Waltham, MA, United States of America; 2 Kirwan Institute for the Study of Race and Ethnicity, Ohio State University, Columbus, OH, United States of America; 3 Knowlton School of Architecture, Ohio State University, Columbus, OH, United States of America; 4 Division of Epidemiology and Community Health, School of Public Health, University of Minnesota, Minneapolis, MN, United States of America; German University in Cairo, CZECH REPUBLIC

## Abstract

In the 1930’s, the Home Owner Loan Corporation (HOLC) drafted maps to quantify variation in real estate credit risk across US city neighborhoods. The letter grades and associated risk ratings assigned to neighborhoods discriminated against those with black, lower class, or immigrant residents and benefitted affluent white neighborhoods. An emerging literature has begun linking current individual and community health effects to government redlining, but each study faces the same measurement problem: HOLC graded area boundaries and neighborhood boundaries in present-day health datasets do not match. Previous studies have taken different approaches to classify present day neighborhoods (census tracts) in terms of historical HOLC grades. This study reviews these approaches, examines empirically how different classifications fare in terms of predictive validity, and derives a predictively optimal present-day neighborhood redlining classification for neighborhood and health research.

## Introduction

The practice of redlining is an example of a racist federal policy that has redistributed resources from historically disadvantaged neighborhoods and population groups towards affluent neighborhoods and Whites, thus reinforcing racial residential segregation and inequality of opportunity [1]. The term “redlining” stems from the neighborhood maps that were drafted by the federally-funded Home Owners’ Loan Corporation (HOLC). HOLC was created in the 1930s as part of the New Deal to assist struggling homeowners. HOLC tasked local officials to draft neighborhood maps that captured variation in default risk across neighborhoods. These officials assigned neighborhoods to one of four color-coded letter grades and labels summarizing their risk assessment: D = “hazardous” (red), C = “definitely declining” (yellow), B = “still desirable” (blue), and A = “best” (green). “A” ratings were assigned to affluent White neighborhoods, while D ratings were assigned to neighborhoods that had a greater share of Black, lower class, or immigrant residents. Virtually all black families lived in D rated areas [2, 3].

HOLC maps reflected pervasive racism in the housing sector prevalent long before the maps were drawn [1, 3], but HOLC took to an unprecedented scale the use of data to facilitate and justify racist appraisal and underwriting practices, thereby formalizing and legitimizing discrimination in the real estate sector and housing policy for subsequent decades [1]. The maps HOLC drew were shared in the real estate sector, notably with the Federal Housing Administration, which drew their own neighborhood maps that influenced the provision of mortgage insurance [2, 4]. Billions of federally guaranteed real estate loans were then channeled into building primarily white affluent neighborhoods, limiting investment in minority neighborhoods, limiting access to homeownership for persons of color, and increasing racial residential segregation [1].

HOLC maps contributed to redirecting the flow of credit from redlined areas, where Black families lived, to greenlined areas where White families lived and from which Black families were excluded [1]. Unequal access to credit facilitated wealth accumulation in White, suburban areas, and disinvestment in Black urban communities. Recent studies show enduring causal effects of HOLC maps on homeownership, home values and racial residential segregation [1, 2, 4]. Furthermore, redlining may have reinforced inequities in access to healthy neighborhood environments more generally, including reduced access to greenspace [5–7]. By promoting the spatial concentration of affluence and disadvantage, as well as racial/ethnic residential segregation, HOLC ratings may have inflicted lasting damage to redlined communities while adding to the privilege of affluent, white communities. These inequities still affect intergenerational economic mobility [4] and social determinants of health [8, 9].

A small but rapidly growing number of studies has examined the long-term health effects of redlining. Neighborhood HOLC ratings have been associated with present-day firearm assaults and violent crimes [10]; preterm birth and other birth outcomes [8, 11], self-rated health [12] and asthma-related emergency department visits [13]. Krieger and colleagues [9] find a substantially elevated risk of late stage at diagnosis among men with lung cancer residing in areas that had been redlined in the past even after adjusting for present-day neighborhood conditions.

One challenge each of these studies faces is how to link historical HOLC rating data to present day individual health outcomes. Existing studies have taken different approaches. None of them has systematically examined how to best classify present-day neighborhoods, i.e., census tracts, in terms of historical HOLC ratings. In this study, we examine different approaches for classifying census tracts in terms of historical HOLC ratings, derive statistically optimal classifications, compare their performance to classifications used in previous research, and propose a simple, predictively optimal classification that can be widely adopted and applied to HOLC rated areas across the US.

Linking HOLC rated areas to census tracts is complicated by the fact that present-day census tracts are generally not nested within HOLC-rated areas, and HOLC-rated areas are generally not nested within census tracts. HOLC areas can cross multiple census tracts, and census tracts can cross multiple HOLC areas. We find that among 14,639 tracts intersecting non-tangentially with HOLC-rated areas, 60% of tracts intersect with multiple HOLC areas that have two, three, or four different grades.

Given that census tracts often intersect with different HOLC-rated areas, classifying tracts in terms of historical HOLC ratings is not straightforward, and there exists no agreed upon method for accomplishing this task. If a census tract intersects with two different rated HOLC areas, how should we classify it? Based on the rating covering the larger portion of the tract? Can the rating covering a smaller portion of the tract be ignored? Does a tract that is 55% covered with A-rated area and 45% covered with D rated area belong to the same class as a tract that is 95% covered with A-rated area and 5% covered with D rated area? We address these and related questions below.

To our knowledge, this is the first paper to empirically examine different approaches to classifying 2010 census tracts in terms of HOLC grades and to develop predictively optimal classifications for linking neighborhood redlining to present day health outcomes. We use a machine learning approach that provides us with both a yardstick to evaluate features of classifications in terms of predictive validity and to derive a predictively optimal classification, i.e., a classification that explains variation in present day census tract outcomes. More generally, this work also illustrates how machine learning can be used in the social sciences to optimize assignment or classification processes that are sometimes performed in an ad-hoc manner.

While previous work has focused on single health outcomes and data for specific states or cities, we employ a total of 17 census tract outcomes to explore the properties of different classifications across all US census tracts that are covered (non-tangentially) by HOLC-rated areas. We consider both health outcomes and social determinants of health that mediate some of the health effects of redlining. And, our census tract HOLC classification data is publicly available on our project website at data.diversitydatakids.org/dataset/holc_census_tracts-1930s-home-owner-loan-corporation—holc—neighborhood-grades-for-u-s—census-trac.

We now review approaches taken to link HOLC ratings to contemporary census tracts in studies examining health effects of redlining and examine the assumptions involved in deriving different classifications. We then use a machine learning approach to identify features of classifications that enhance predictive validity and derive predictively optimal classifications.

### Previous approached to classifying census tracts based on HOLC ratings

Existing research on health outcomes of redlining uses census tracts to link contemporary health data to historical HOLC area ratings [8–10, 12, 13]. Census tracts capture important features of individual residential context or neighborhoods [14]. Five out of six studies on health outcomes reviewed for this study used census tracts to link historical HOLC ratings to present day health outcomes.

Two studies have calculated the proportion of census tract area covered by different HOLC grades and used these proportions to estimate the association between HOLC ratings and present day outcomes [10, 12]. This approach is efficient for multivariate but inefficient for descriptive analyses. For any given tract, up to four variables (proportion rated A, B, C, D) have to be considered jointly to identify its rating status, which complicates descriptive analyses and mapping. Four separate maps or map layers would be required to visualize HOLC ratings at the census tract level for a given area.

After calculating the proportion of census tract area covered by different HOLC ratings, other studies took the additional step of classifying tracts as either A, B, C, or D rated [8, 9, 13]. In the sample of tracts studied by Krieger et al. [9], 16% of tracts were fully enclosed by an area with a single HOLC rating and assigned that rating. 54% of tracts were covered by different rated HOLC areas. The remaining 29% were treated were categorized as “no grade assigned”, because less than 50% of the tract area was covered by HOLC-rated areas [9]. The classification preserves the intuition behind the original HOLC ratings, assigning one grade to a neighborhood, which facilitates descriptive analyses and mapping. However, it also discards information that may be of predictive value, disregarding secondary ratings and disregarding rating information for tracts covered by multiple different rated HOLC areas, or not sufficiently covered by HOLC-rated areas.

Nardone et al link census tracts to HOLC ratings by assigning tracts to the rating of the HOLC area that contained the census tract centroid [13]. Because census tracts and HOLC areas have irregular shapes, and one census tract can intersect with multiple HOLC areas that have different ratings, it is not guaranteed that this approach assigns the HOLC rating to a tract that covers the largest portion of the tract’s area.

### Assigning HOLC ratings to census tracts

Previous studies took different approaches when classifying census tracts in terms of HOLC ratings. Here, we identify key empirical choices (to be) made when classifying tracts and subsequently explore how they affect the predictive validity of the resulting classification.

#### Size of area covered

**Fig 1:**
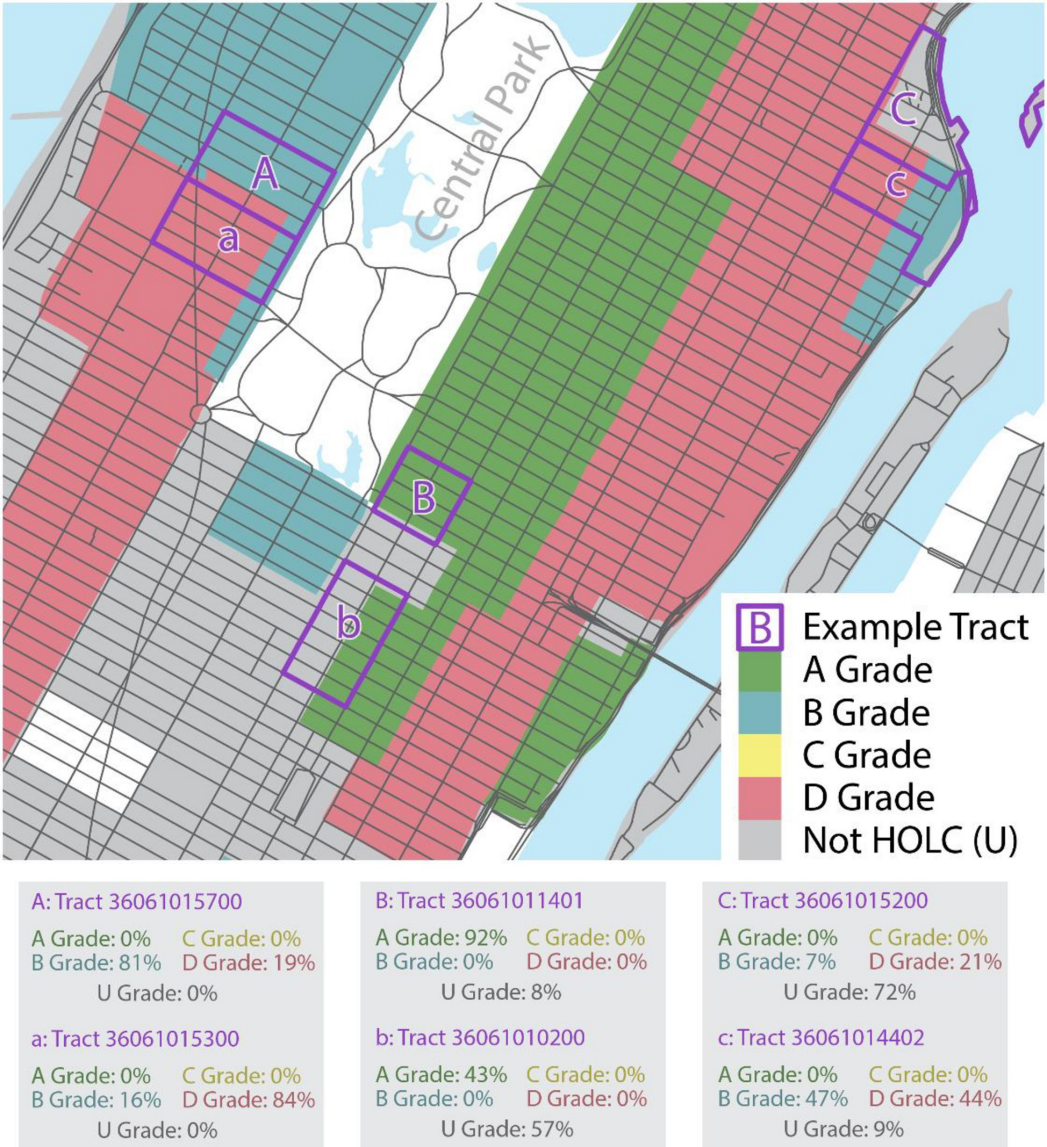
Census tracts and HOLC-rated polygons in Manhattan, New York City. Note: The map image was created using the 2019 TIGER/Line Shapefiles (machine readable data files) / prepared by the U.S. Census Bureau and HOLC rating data published by the Digital Scholarship Lab under a Creative Commons Attribution-NonCommercial-ShareAlike 4.0 International License (https://creativecommons.org/licenses/by-nc-sa/4.0/).

Should classifications account for the size of the area covered by a given rating? For example, in Fig 1, Tract *B* is 92% covered by B ratings, while tract *b* is 43% covered by B ratings. Should we assign Tract *B* and *b* to the same or different classes?

#### Single vs. multiple ratings

We found 60% of tracts that are non-tangentially covered by HOLC-rated areas are covered by two or more ratings. When a census tract is covered by more than one rating, should we consider only the rating covering the largest part of the census tract, or should we also consider additional ones? For example, in Fig 1, tracts *A* and *a*, as well as *C* and *c* overlap with HOLC areas that have different ratings. Tract *c*’s area is 47% covered by B and 44% by D rated areas. Should we focus on the B rating and ignore the D rating, or consider both?

#### Absolute vs. relative size

For tracts intersecting with different rated areas, do we need to consider the absolute or just the relative size of the area covered by the different rated areas? For example, tract *C* is 21% covered by D and 7% covered by B rated area, while tract *a* is 84% covered by D and 19% covered by B rated area. We could assign them to the same two-rating class, e.g., “Mainly D, some B”, based on ordering the rankings for each tract in terms of the area covered. If we accordingly just consider the relative sizes of the areas for each tract, both *C* and *a* would belong to the same class, because, for each tract, D-rated areas cover more than A-rated areas. We could also assign them to different classes, if our classification algorithm would consider the absolute size of the area covered by the different ratings. The D-rated area covers a much larger proportion of tract *a* compared to *C* (84% vs. 21%) and leveraging that information might prove consequential for predicting the long-term effects of redlining.

#### “Unrated” as a separate grade

Do tracts that are only partially covered by one or more HOLC areas belong to the same or different classes? For example, if Tract *B* is 92% covered by A ratings and 8% covered by unrated area and Tract *b* is 43% covered by A rated area and 57% covered by unrated area, should they both be rated A? In exploratory analyses, we observed that tracts with larger portions of unrated area have on average better outcomes, perhaps because some of these unrated areas were later incorporated into affluent suburbs. Jacoby et al [10] find unrated areas to have higher prevalence of firearm assaults (compared to A-rated areas). We could therefore either include un-ratedness (U) as its own category or treat it as missing at random.

#### Minimal coverage

Some tracts are only marginally covered by HOLC areas. Is there a minimal threshold we should impose when classifying tracts in terms of HOLC ratings? For example, tract *C* is 28% covered by D and B rated area, and 72% covered by unrated area. Should we discard tracts that are only, e.g., 5% covered by rated area?

### Testing the predictive validity of different classifications using cross-validation

Like the studies reviewed above, we first calculated–for every census tract that intersects non-tangentially with HOLC rate area–the proportion of the tract that is A, B, C, or D rated, or unrated (U). Based on these proportions, we generated 54 different classifications and datasets that independently vary each of the five features described in the preceding section. We then tested how well each of these classifications explains variation in six present day health and socioeconomic outcomes using five-fold cross-validation in combination with Ordinary Least Squares (OLS) regression [15]. We repeated the analysis on seven different datasets that exclude tracts at different rates based on a threshold for minimum area that needs to be covered by HOLC ratings. With the data generated from the cross-validation experiments, we study which features of the classifications impact their predictive validity and how, identify predictively optimal classifications, and we identify a parsimonious 10-class classification for applied research. Finally, we compare the predictive validity of the classifications thus identified to classifications used in previous research.

## Materials and methods

### Data

#### Shapefiles

We rely on HOLC rating maps published by Nelson et al. [16] and 2019 TIGER/Line Shapefiles for census tracts, area hydrography, and core-based statistical areas from the Census Bureau [17–19]. We start with selecting all census tracts within the 143 Core Based Statistical Areas that have a HOLC rating, excluding any census tract that has zero total population. This limits the pool of census tracts from approximately 73,000 to 44,730. We exclude water areas from the census tract and HOLC polygon areas in the study area. We combine census tract and HOLC polygons using the ArcMap Union tool and calculate the proportion of census tract area that is covered by A, B, C, D and U grades. We initially include all census tracts that are at least 1% covered by either A, B, C, or D rated areas, which are 14,639 census tracts prior to additional exclusions due to missing outcome data.

#### Census tract data

Census tract level data on life expectancy is from the Centers for Disease Control (CDC) U.S. Small-area Life Expectancy Project (USALEEP) for the period from 2010–2015 [20, 21]. The RWJF-CDC 500 Cities project provides census tract-level estimates of healthy behaviors and conditions based on spatially interpolated survey data [22, 23]. We used the following prevalence indicators, measured in 2015 and for adults aged 18 years or older: having five or more drinks (men) or four or more drinks (women) on an occasion in the past 30 days, having smoked in their lifetime or currently smoke every day or some days, no leisure-time physical activity in the past month, having a body mass index equal to or greater than 30, having ever been diagnosed with or currently having asthma, having ever been diagnosed with any type of cancer (besides skin cancer), having ever been diagnosed with angina or coronary heart disease, having ever been diagnosed with or currently having diabetes, mental health not good for 14+ days, and physical health not good for 14+ days.

The Opportunity Atlas includes census tract level indicators of intergenerational mobility [14, 24]. We selected the following metrics, measured in 2015: Mean household income rank at age 35 for children with parents at the 50th percentile of the parent income distribution; probability of living in a low poverty census tract at age 35 for children with parents at the 50th percentile of the parent income distribution. Additionally, we used the two versions of this metric calculated for children with parents at the 25^th^ percentile of the parent income distribution. Finally, we also used an indicator ranking in the top 20% of the household income distribution at age 35 for children with parents at the 25th percentile of the parent income distribution.

The Child Opportunity Index (COI) 2.0 is a composite index calculated from 29 indicators measuring neighborhood features that matter for children’s healthy development [25, 26]. We used 2015 nationally normed Child Opportunity Scores from the COI 2.0 database, which rank neighborhoods on a scale from 1 (lowest) to 100 (highest).

Opportunity Atlas and COI 2.0 data are available for nearly all US census tracts while data from the 500 Cities database is only available for census tracts in the 500 largest US cities, leading to a loss of some HOLC classified census tracts in analyses using 500 Cities outcomes.

### Methods

#### Classifications

Using the procedure outlined above, we obtained the percentage tract area that is covered with A, B, C, and D rated areas, and unrated (U). For each tract, the sum of A, B, C, D and U is equal to 100%. A tract can be 100% covered by one type of rating (except U), or any combination of ratings, e.g., 80% A and 20% B.

We then construct six sets of classifications, numbered from 1 to 6. Each set consists of five individual classifications, numbered from 1 to 5. The sets differ in terms of how much detail of the original percentages (measuring the percentage of the tract area covered by a given rating) they preserve and how many ratings they combine. For example, for classification set 1, we coarsen each percentage coverage variable into 10 percentage point wide bins, creating a variable with 10 ordered values, where 1 corresponds to percentages equal to zero and less than 10, 2 corresponds to percentages equal to or greater than 10, but less than 20, and so forth. More specifically:

Set 1. Each percentage variable is coarsened into 10 percentage point wide bins, resulting in variables with 10 ordered values, where 1 = [0%,…,10%), 2 = [10%,…,20%), …, 10 = [90%,…,100%].
Set 2. Each percentage variable is coarsened into 20 percentage point wide bins, resulting in variables with 5 ordered values, where 1 = [0%,…,20%), 2 = [20%,…,40%), …, 5 = [80%,…,100%].
Set 3. Each percentage variable is coarsened into 25 percentage point wide bins, resulting in variables with 4 ordered values, where 1 = [0%,…,25%), 2 = [25%,…,50%), …, 4 = [75%,…,100%].
Set 4. Each percentage variable is coarsened into 33.3 percentage point wide bins, resulting in variables with 3 ordered values, where 1 = [0%,…,33.3%), 2 = [33.3%,…,66.6%), 3 = [66.6%,…,100%].
Set 5. Each percentage variable is coarsened into 50 percentage point wide bins, resulting in variables with 2 ordered values, where 1 = [0%,…,50%), 2 = [50%,…,100%].

The classifications within each set differ in terms of how many different ratings per tract they consider. 1-rating classifications only consider the most important rating, i.e., the rating covering the largest area for the tract. A possible value for the 1-rating classification in set 1, is A8, corresponding to a tract that is between 70% and 80% covered with an A-rated HOLC area. A two-rating classification considers the most important and the second most important rating. A possible value for the two-rating classification in set 2 would be A8-B1, i.e., a tract 70–80% covered with A and 0–10% covered with B.

We also created a sixth set of classifications. The first five preserve information about the absolute size of the area covered by different ratings. Therefore, a tract that is 85% A and 15% B may be assigned to a different class than a tract that is 55% A and 45% B. (That’s true for classification sets 1 to 4, but not 5 they would be assigned the same value, A2-B1.) The sixth set of classifications only considers the ranking of areas in terms of how much of a tract’s area they cover.

Set 6. We create five variables, where the first one contains the most important rating, e.g., A, the second contains the second most important rating, …, and the fifth variable contains the fifth and least important rating. As before, we create five different classifications that use between one and five different ratings.

To test whether excluding U improves our ability to predict outcomes, we re-create each of the aforementioned classifications excluding U as a separate rating category. We recalculate the total area for each tract as the total rated tract area, i.e., the tract area that is covered by HOLC areas. We then recalculate the tract area percentages relative to this new denominator. Using these percentages, we recreate the six sets, each of which now contains only four classifications. This yields a total of 54 classifications. Table 1 lists the classifications, the number of distinct classes per classification and an example value.

**Table 1:** 54 classifications of 2010 census tracts in terms of historical HOLC rating status.

	Rank-ordered (set 6)			50 percentage points (set 1)		33 percentage points (set 2)		25 percentage points (set 3)		20 percentage points (set 4)		10 percentage points (set 5)	
**Includes unrated tract portion?^1^**	**Number of ratings considered** ^**2**^												
		*Classes* ^*3*^	*Example* ^*4*^	*Classes*	*Example*	*Classes*	*Example*	*Classes*	*Example*	*Classes*	*Example*	*Classes*	*Example*
**No**	**1**	4	C	8	C0	12	C1	12	C1	16	C1	29	C3
**No**	**2**	*16*	*C-B*	28	C0-B0	52	C1-B0	58	C1-B1	77	C1-B1	189	C3-B3
**No**	**3**	40	C-B-A	63	C0-B0-A0	115	C1-B0-A0	146	C1-B1-A0	195	C1-B1-A1	473	C3-B3-A2
**No**	**4**	64	C-B-A-D	106	C0-B0-A0-D0	178	C1-B0-A0-D0	233	C1-B1-A0-D0	308	C1-B1-A1-D0	679	C3-B3-A2-D1
**Yes**	**1**	5	C	10	C1	15	C1	15	C2	20	C2	40	C5
**Yes**	**2**	24	C-U	44	C1-U0	89	C1-U0	102	C2-U0	137	C2-U0	342	C5-U1
**Yes**	**3**	*84*	*C-U-D*	139	C1-U0-D0	246	C1-U0-D0	335	C2-U0-D0	465	C2-U0-D0	1154	C5-U1-D1
**Yes**	**4**	194	C-U-D-B	318	C1-U0-D0-B0	508	C1-U0-D0-B0	684	C2-U0-D0-B0	904	C2-U0-D0-B0	1988	C5-U1-D1-B1
**Yes**	**5**	289	C-U-D-B-A	455	C1-U0-D0-B0-A0	661	C1-U0-D0-B0-A0	858	C2-U0-D0-B0-A0	1084	C2-U0-D0-B0-A0	2138	C5-U1-D1-B1-A0

^1^If yes, classifications include unrated portion of census tracts, i.e., the portion not covered by an A, B, C, or D rated polygon, as a separate grade (“U”).

^2^The number of ratings considered when classifying tracts. 1 = only HOLC polygon covering the largest area of the tract is considered, 2 = the polygons covering the largest and second largest area of a tract are considered, etc.

^3^The number of distinct classes into which tracts are classified.

^4^An example labeled class value for a given classification.

#### Five-fold cross-validation

We use five-fold cross-validation and Ordinary Least Squares (OLS) regression to quantify the predictive validity of different classifications [15]. Our goal is to study the properties of different classifications across different health outcomes and their underlying social determinants. We use three global socio-economic and three global health metrics: life expectancy (USALEEP), mental health (500 Cities), physical health (500 Cities), household income rank (children with parents at median of parent income distribution), residing in a low poverty neighborhood (median), and COI 2.0. Associations between HOLC ratings and three of these metrics have been previously documented for physical health [12], residing in a low poverty neighborhood [4], and household income rank [4]. The COI 2.0 includes several social and environmental determinants of health that previous research has found to be associated with redlining, including access to green-space and neighborhood socio-economic composition. [2, 5] Life expectancy and mental health are strongly associated with neighborhood social determinants of health that are in turn likely to be affected by HOLC ratings.

For every outcome, we start with a dataset that has non-missing data on that outcome and standardize the dependent variable. We randomly divide the data into five folds, numbered from 1 to 5. We then iterate over classifications j from 1 to 54. For each classification, we iterate over five folds. For each fold k, we perform the following operations:

- We hold out fold k as the test dataset and the remaining folds constitute the training data.
- In the training data, we run an OLS regression of a given outcome, transformed to z-scores by subtracting the outcome mean and dividing by the outcome standard deviation, on all classes of the given classification (omitting one class as the reference group).
- We then combine the classification information in the test data with the regression coefficient obtained from the training data to predict outcome values in the test data.
- Using the test data, we calculate the observation-specific prediction error as the differences between observed minus predicted outcome.
- From these observation-specific errors, we calculate the mean squared error (MSE) of the prediction in the test data, which is simply the average of the squared prediction errors across all observations in the test data.
- We record this average MSE for the given fold.

After repeating this process for each of the five folds, we average the resulting five MSEs into a single MSE for a given classification and outcome. A smaller MSE indicates better predictive validity. We run this algorithm once for each of the 54 for classifications and six outcomes.

We perform the aforementioned cross-validation analysis on seven different datasets. The datasets use different thresholds for excluding tracts that are only partially covered by graded HOLC polygon, including tracts that are minimally 1%, 5%, 10%, 15%, 25%, 33%, and 50% covered by HOLC graded areas. Altogether, this produces a dataset of (54 classifications x 6 outcomes x 7 datasets with different thresholds =) 2,268 averaged, cross-validated MSEs.

We graphically explore the bivariate association between MSEs and different features of the classifications and conduct multivariate OLS regression analyses regressing MSEs on variables capturing the main features of the experiments and classifications.

#### Deriving predictively optimal and parsimonious classifications

Based on the data generated by the cross-validation experiments, we identified the predictively optimal classification, i.e., the classification with the lowest average test MSE across all experiments. We also identified a predictively optimal parsimonious classification, which was the classification with the fewest classes that achieved an average test MSE close to the predictively optimal classification. We further collapsed this parsimonious classification to a 10-class classification that is easy to use in applied research. We combined classes that are both small and have similar average outcome levels. Average outcome levels were calculated by first standardizing the outcomes used in the cross-validation analysis. We then multiplied the physical and mental health measures by -1 so that for each outcome, a higher value equals a better outcome. We then averaged across the six outcomes. Combining outcomes in this manner is justified by their high degree of intercorrelation (alpha = 0.93).

#### Comparing novel and published classifications

Finally, we examine the predictive performance of three novel classifications identified through the cross-validation experiments, and compare them to classifications/approaches taken by the studies reviewed above. For these analyses, we use a different set of outcomes not used in the cross-validation analysis: smoking, binge drinking, no leisure time physical activity, obesity, diabetes, asthma, coronary heart disease, and cancer from the 500 Cities data; and from the Opportunity Atlas, we use household income rank (children with parents at the 25^th^ percentile of the parent income distribution), residing in a low poverty neighborhood (25^th^ percentile), and household income in top 20% (25^th^ percentile).

This study was deemed exempt (not human subjects research) by the Brandeis University Committee for Protection of Human Subjects, IRB Protocol #17118.

Replication materials have been published at github.com/diversitydatakids/plos_one_connecting_past_to_present_replication_materials.

## Results

**Fig 2:**
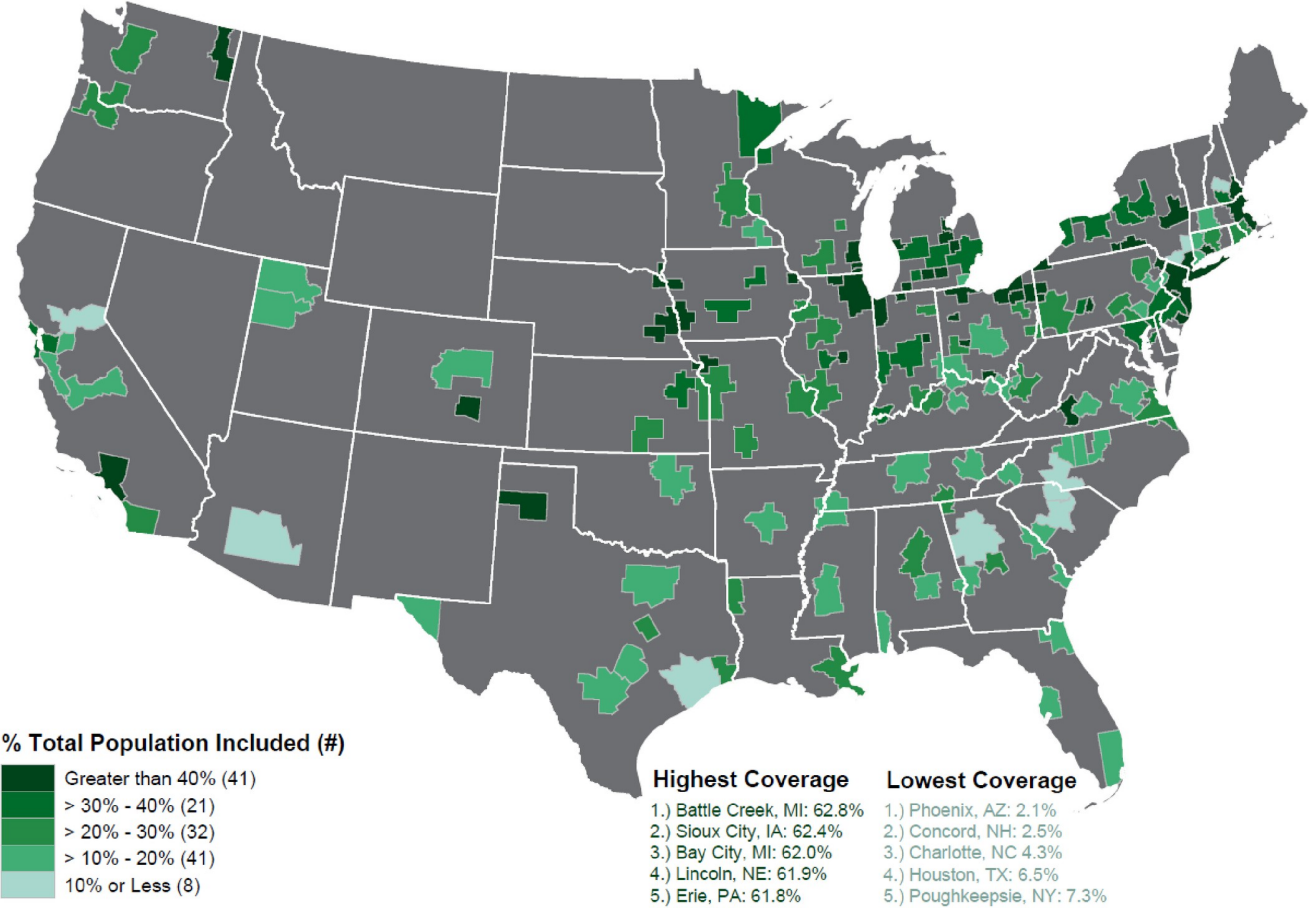
Core based statistical areas. Percentage of population in census tracts with 1% or more HOLC rating coverage. Note: The map image was created using the 2019 TIGER/Line Shapefiles (machine readable data files) / prepared by the U.S. Census Bureau and HOLC rating data published by the Digital Scholarship Lab under a Creative Commons Attribution-NonCommercial-ShareAlike 4.0 International License (https://creativecommons.org/licenses/by-nc-sa/4.0/).

HOLC residential security maps were created for 239 cities, which had populations that exceeded 40,000 people. As Fig 2 illustrates, the Core Based Statistical areas with the highest coverages are mostly in mid-sized rust belt cities which haven’t experienced significant growth or sprawl. Legacy cities such as New York, Chicago, and Los Angeles have higher coverages of residents today living in a HOLC graded neighborhoods mostly due to their size and density. Many cities with the least HOLC coverage are in regions which have experienced significant growth since the 1970’s in the Sun Belt or in western metros like Phoenix, Houston, Atlanta, and Raleigh-Durham.

Table 2 reports descriptive statistics for tracts included and excluded in the analysis. Only tracts that are at least 1% covered by HOLC ratings are included in the analysis, which yields a sample of 14,639 tracts. Excluded tracts are tracts that are not covered by HOLC-rated areas or less than 1% covered by HOLC-rated areas. Census tracts covered by historical HOLC ratings have, on average, worse outcomes than tracts that are not covered. For example, life expectancy in HOLC-rated tracts is one year lower than in tracts with no HOLC ratings. Among included tracts, we observe that A ratings cover the smallest area, 4.2% of included tracts, while C ratings have the largest coverage, 30.7 percent. Among included tracts, 31.9% of the area is covered by unrated area.

**Table 2:** Descriptive statistics.

	Excluded Tracts			Included Tracts		
	*Mean*	*SD*	*N*	*Mean*	*SD*	*N*
*Proportion of tract covered by HOLC polygon*						
Proportion covered by A-rated polygon	0.00	0.02	58,417	4.19	14.76	14,639
Proportion covered by B-rated polygon	0.00	0.02	58,417	13.91	26.09	14,639
Proportion covered by C-rated polygon	0.00	0.03	58,417	30.72	35.01	14,639
Proportion covered by D-rated polygon	0.00	0.02	58,417	19.32	31.64	14,639
Proportion unrated	100.00	0.05	58,417	31.86	32.60	14,639
*Dependent variables*						
Life expectancy*	78.50	3.75	52,367	77.47	4.77	13,295
Physical health*	12.12	3.64	15,678	13.68	4.59	11,284
Mental health*	12.59	3.15	15,678	13.86	3.62	11,284
Binge drinking	17.55	3.76	15,678	17.77	4.64	11,284
Cancer	5.64	1.92	15,678	5.30	1.49	11,284
Asthma	9.19	1.44	15,678	10.43	2.12	11,284
Coronary heart disease	5.61	2.02	15,678	6.11	2.13	11,284
Smoking	17.04	5.23	15,678	19.63	6.76	11,284
Diabetes	10.02	3.60	15,678	11.95	4.88	11,284
Limited physical activity	23.70	8.32	15,678	28.00	10.02	11,284
Obesity	29.01	6.95	15,678	31.68	9.54	11,284
COI 2.0*	52.19	27.20	57,583	37.22	30.84	14,630
Household income rank (p50)*	50.61	5.79	57,374	46.73	8.13	14,580
Residence in low poverty neighborhood (p50)*	48.95	17.95	57,366	41.87	16.81	14,575
Household income rank (p25)	43.49	6.68	57,374	40.41	8.18	14,580
Household income in top 20% (p25)	12.81	7.79	57,374	11.40	9.02	14,580
Residence in low poverty neighborhood (p25)	44.36	19.52	57,366	36.77	17.54	14,575

Note: Tracts with 1% or more HOLC rating coverage are included in the analysis

*Dependent variable included in cross-validation analysis.

Table 3 reports the results from the multivariate OLS regression of the averaged, cross-validated MSEs from the classification experiments on the features of the classifications. The average MSE across all 2,628 experiments is 0.89 and the standard deviation is 0.05. The coefficient estimates and standard errors in column 6 are based on a regression that includes all features simultaneously. The remaining columns report results from regressions including one feature at a time. Comparing coefficient estimates for a given feature across columns, we find very similar results. The features of the data and classifications used were experimentally manipulated and therefore largely uncorrelated with each other. We therefore focus on column 6.

**Table 3:** OLS regression coefficients from regressions of cross-validated mean squared prediction errors on experiment features, based on 2,628 cross-validation experiments.

	*(1)*	*(2)*	*(3)*	*(4)*	*(5)*	*(6)*
2 ratings (Ref.)						
1 rating	0.029***					0.029***
	(0.003)					(0.001)
3 ratings	0.008**					0.008***
	(0.003)					(0.001)
4 ratings	0.027***					0.027***
	(0.003)					(0.001)
5 ratings	0.057***					0.047***
	(0.003)					(0.002)
Rank ordered, set 6 (Ref.)						
50% bins, set 5		0.004				0.004*
		(0.003)				(0.002)
33% bins, set 4		0.015***				0.015***
		(0.003)				(0.002)
25% bins, set 3		0.017***				0.017***
		(0.003)				(0.002)
20% bins, set 2		0.022***				0.022***
		(0.003)				(0.002)
10% bins, set 1		0.055***				0.055***
		(0.003)				(0.002)
Exclude unrated (Ref.)						
Include unrated			0.026***			0.019***
			(0.002)			(0.001)
COI 2.0 (Ref.)						
Household income rank				0.032***		0.032***
				(0.003)		(0.002)
Life expectancy				0.077***		0.077***
				(0.003)		(0.002)
Low pov. neighborhood				0.022***		0.022***
				(0.003)		(0.002)
Mental health				0.043***		0.043***
				(0.003)		(0.002)
Physical health				0.074***		0.074***
				(0.003)		(0.002)
Threshold = 5% (Ref.)						
Threshold = 1%					0.001	0.001
					(0.003)	(0.002)
Threshold = 10%					0.000	0.000
					(0.003)	(0.002)
Threshold = 15%					0.001	0.001
					(0.003)	(0.002)
Threshold = 25%					0.002	0.002
					(0.003)	(0.002)
Threshold = 33%					0.003	0.003
					(0.003)	(0.002)
Threshold = 50%					0.003	0.003
					(0.003)	(0.002)
Constant	0.869***	0.867***	0.873***	0.846***	0.886***	0.796***
	(0.002)	(0.002)	(0.001)	(0.002)	(0.002)	(0.002)
Observations	2,268	2,268	2,268	2,268	2,268	2,268
R-squared	0.16	0.15	0.08	0.38	0.00	0.74

Note:

*** p<0.001

** p<0.01

* p<0.05.

First, classifications that use two ratings, i.e., the ratings covering the most and second-most area of a tract, have the lowest MSEs on average. Adding potential third or fourth ratings reduces predictive validity. Compared to two ratings, using only one rating increases MSEs by more than half a standard deviation. Second, when assigning a tract covered by more than two ratings to a specific class, it is sufficient to consider the relative size of the area covered by different ratings (set 6) rather than the absolute size in percent (sets 1–5). In other words, we would assign a tract that is 90% covered by A and 10% covered by B rated area to the same class as a tract that is 51% covered by A and 49% covered by B rated area. Third, classifications that included information on the unrated tract portion as a separate grade perform worse.

Altogether, this suggests that, in general, more detailed classifications perform worse. The exception to this pattern is that rather than only considering the most important rating, adding information on the second most important rating improves predictive performance.

Fourth, we do not observe substantial differences across experiments that limit the number of tracts included based on the total percentage of the tract area that is A, B, C, or D rated areas. Varying the threshold for exclusion between 1% and 50% minimal coverage, we observe no substantial differences in average MSE. Among the tested thresholds, the 5% cutoff has the lowest average MSEs, suggesting that it is justified to include tracts that are at least 5% covered by HOLC-rated areas in empirical analyses. Fifth, among the different outcomes used in the cross-validation analysis, the index of neighborhood conditions conducive to health child development, COI 2.0, yields the lowest MSE on average.

We also examined if the patterns observed in Table 3 vary across dependent variable by stratifying the MSE dataset and rerunning the OLS regression reported in column 6 of Table 3 separately for each dependent variable. The pattern is essentially identical across dependent variables Table 1 in S1 Appendix). The only notable difference is that for residence in a low poverty neighborhood, classifications including unrated area as a separate rating do not perform worse on average.

**Fig 3:**
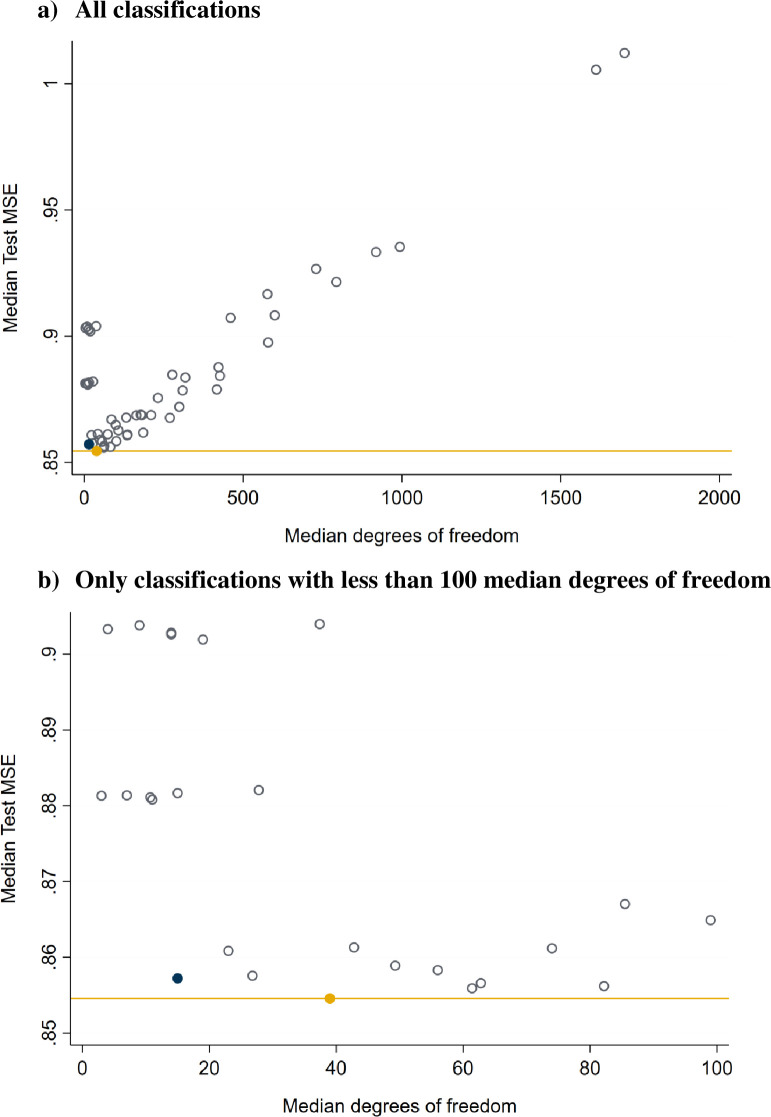
Median MSE across experiments for each of the 54 classifications tested, plotted against the average degrees of freedom used by a given classification. A) All classifications. b) Only classifications with less than 100 median degrees of freedom. Note: The yellow horizontal line is drawn at the minimum averaged MSE across the 54 different classifications. The yellow circle marks the best fitting classification. The blue circle is the most parsimonious classification that is closest to the minimum averaged MSE.

For Fig 3A, we averaged the cross-validated MSEs across all 42 experiments for a given classification (6 outcomes x 7 datasets with varying thresholds for inclusion) and plotted these against the median degrees of freedom these classifications use. The median degrees of freedom are slightly lower than the number of distinct classes listed in Table 1, because–especially for more detailed classifications—not all theoretically possible classes exist empirically. The horizontal yellow line marks the minimum averaged MSE obtained by one of the classifications.

Median MSEs decline steeply as classifications become more detailed initially, reaching a minimum at 39 degrees of freedom. Thereafter, MSEs increase linearly as a function of degrees of freedom. Increasingly detailed classifications result in overfitting in the training data and declining predictive performance in the test data. Many associations that increasingly detailed classifications would find in the training data are idiosyncratic to the training folds and do not replicate in the testing folds.

Fig 3B shows a subset of the data shown in Fig 3A, focusing only on classifications with median degrees of freedom less than 100. Several classifications yield average MSEs close to the minimum, but they differ substantially in terms of the degrees of freedom used. The best performing classification from a prediction standpoint, marked by a solid yellow circle, belongs to set 6 (rank-ordered) and considers the three most important ratings. It uses 39 degrees of freedom, excluding the constant. We have also highlighted the best performing classification that uses the fewest degrees of freedom in the lower left corner of the plot (blue circle), which also belongs to set 6 (rank-ordered) and considers the two most important ratings only. It uses 15 degrees of freedom and performs just marginally worse than the best fitting classification.

We also calculated classification-specific averages MSEs separately by dependent variable. Fig 1 in S1 Appendix visualizes the distribution of the 54 MSEs for each dependent variable. We have highlighted the two classifications identified in Fig 3A and 3B. Both classifications are among the optimally fitting ones for each dependent variable. The pattern differs somewhat for the residing in a low poverty neighborhood outcome, for which the two classifications rank in the top 20 of best fitting classification.

Next, we further simplified the 15 degrees of freedom classification (blue circle in Fig 3A and 3B). The left column of Table 4 shows the original classification with 16 classes, the right column shows a simplified version with 10 classes that is more tractable for explanatory and descriptive research. For each classification, we list the class labels, class sizes (number of census tracts) and average standardized outcome levels. First, we combine all classes that are only or mainly A. Second, we combine mainly C or D classes that have A as their second most important rating; and, we combine mainly C or D rated classes that have some B as their second most important rating. Finally, we combine mainly B rated classes with either some C or some D as their second most important rating. These simplifications come with only a minimal loss of predictive performance (see below).

**Table 4:** Detailed and collapsed rank-ordered, two-rating classification.

*Rank-ordered*, *two ratings*, *detailed*			*Rank-ordered*, *two ratings*, *collapsed*		
*Class label*	*Number of census tracts*	*Average standardized outcome*	*Class label*	*Number of census tracts*	*Average standardized outcome*
1. Only A	223	0.87	Only or mainly A (1–4)	981	0.86
2. Mainly A, some B	544	0.89			
3. Mainly A, some C	171	0.81			
4. Mainly A, some D	43	0.51			
5. Only B	785	0.41	Only B (5)	785	0.41
6. Mainly B, some A	566	0.61	Mainly B, some A (6)	566	0.61
7. Mainly B, some C	1,388	0.31	Mainly B, some C or D (7–8)	1,562	0.29
8. Mainly B, some D	174	0.20			
9. Only C	2,760	0.09	Only C (9)	2,760	0.09
10. Mainly C, some A	199	0.39			
11. Mainly C, some B	1,800	0.11			
12. Mainly C, some D	1,840	-0.30	Mainly C, some D (12)	1,840	-0.30
13. Only D	2,156	-0.31	Only D (13)	2,156	-0.31
14. Mainly D, some A	47	0.44			
15. Mainly D, some B	193	-0.07			
16. Mainly D, some C	1,750	-0.47	Mainly D, some C (16)	1,750	-0.47
			Mainly C or D, some A (10 & 14)	246	0.40
			Mainly C or D, some B (11 & 15)	1,993	0.09

Next, we examined the predictive validity of three classifications identified by cross-validation experiments and two approaches/classifications used in existing research. We use outcomes that were not used during the cross-validation experiments and when deriving the optimal classifications. Based on the results reported in Table 3, we used all tracts that are at least 5% covered by HOLC ratings. We ran bivariate OLS regression of a given outcome on a given classification and recorded R-squared statistics from each regression. We also recorded the root mean squared error and adjusted R-squared statistics (available upon request), which show a pattern that is very similar to the pattern for overall R-squared statistics.

The first column of Table 5 is based on regressions of a given outcome on the proportion of a tract that is covered by B, C, or D rated areas, similar to the one used by McClure et al. [12] and Jacoby et al. [10]. Proportions were calculated using the total rated area as the denominator. The proportion that is rated A is omitted for the model to be identified. The second column reports results using a classification that assigns each tract to either A, B, C, or D, depending on which rating covers the largest area, similar to the one used in the studies by Krieger et al. [8, 9]. The remaining columns report results for the collapsed parsimonious (10 classes), detailed parsimonious (16 classes), and predictively optimal classifications (40 classes) identified in the preceding analyses. The underlying models use between 3 and 39 degrees of freedom, excluding the constant.

**Table 5:** R-squared statistics from OLS regressions of 11 census tract level health and socio-economic outcomes on different census tract HOLC rating measures/classifications.

	Proportions	One rating	Rank-ordered, 2 ratings, collapsed	Rank-ordered, 2 ratings, detailed	Rank-ordered, 3 ratings, optimal
**Degrees of Freedom**	3	3	9	15	39
*Health Outcomes*					
Drinking	0.02	0.02	0.03	0.03	0.04
Cancer	0.12	0.11	0.13	0.13	0.13
Asthma	0.07	0.06	0.07	0.08	0.08
CHD	0.02	0.02	0.03	0.03	0.04
Smoking	0.10	0.09	0.12	0.12	0.13
Diabetes	0.07	0.06	0.08	0.08	0.08
Phys. act.	0.11	0.10	0.12	0.12	0.13
Obesity	0.06	0.05	0.08	0.08	0.09
*Average*	*0*.*07*	*0*.*06*	*0*.*08*	*0*.*08*	*0*.*09*
*Economic outcomes*					
Income rank	0.11	0.10	0.13	0.13	0.14
Income in top 20%	0.11	0.10	0.12	0.12	0.13
Low pov. neighborhood	0.15	0.13	0.15	0.15	0.16
*Average*	*0*.*12*	*0*.*11*	*0*.*13*	*0*.*14*	*0*.*14*

Note: All outcome were standardized using the z-score transformation.

We observe that incremental gains from additional degrees of freedom are relatively small. Comparing the collapsed and optimal classification, the former uses 9 degrees of freedom to explain on average 8.3% (13.3%) of the variation across health (socio-economic) outcomes, while the latter uses 39 degrees of freedom to explain 9.0% (14.3%) of the variation across health (socio-economic) outcomes. In other words, compared to the predictively optimal classification, the collapsed classification uses less than quarter of the degrees of freedom for 90% of the predictive performance relative to the predictively optimal classification. The latter percentage is calculated as 100 x 8.3/9.2.

Compared to the rank-ordered one rating classification which uses three degrees of freedom to explain on average 6.2% (11.1%) of the variation in health (socio-economic) outcomes, the collapsed classification uses six additional degrees of freedom to explain 8.3% (13.3%) of variation, a 34% (20%) improvement in predictive performance for health (socio-economic) outcomes. The collapsed classification also performs better predictively than the approach using proportions, which in turn perform better than the rank ordered one rating classification.

Finally, Table 6 reports the regression coefficients for two outcomes, diabetes diagnoses and household income rank, and two classifications. For both sets of regressions, the reference group is tracts that are only or mainly covered by A-rated areas. This includes tracts that are only covered by A-rated areas, or that are covered A-rated areas and areas with other ratings where the A-rated polygon covers the largest of the rated area.

**Table 6:** OLS regression coefficients and standard errors from regressions of census tract level adult household income ranks (Opportunity atlas) and diabetes diagnoses (500 cities) on two census tract HOLC classifications.

	*Household income rank (p25)*		*Diabetes*	
	Rank-ordered, one rating	Rank-ordered, two ratings (collapsed)	Rank-ordered, one rating	Rank-ordered, two ratings (collapsed)
Only or mainly A (Ref.)				
Only or mainly B	-3.35***		1.76***	
	(0.29)		(0.22)	
Only or mainly C	-5.70***		3.22***	
	(0.27)		(0.20)	
Only or mainly D	-9.28***		4.46***	
	(0.28)		(0.21)	
Only B		-2.47***		2.23***
		(0.38)		(0.28)
Mainly B, some A		-2.16***		0.75*
		(0.41)		(0.31)
Mainly B, some C or D		-4.17***		1.88***
		(0.32)		(0.24)
Only C		-4.29***		3.06***
		(0.29)		(0.22)
Mainly C, some D		-8.44***		4.21***
		(0.31)		(0.23)
Only D		-8.83***		4.03***
		(0.30)		(0.22)
Mainly D, some C		-10.11***		5.21***
		(0.31)		(0.23)
Mainly C or D, some A		-3.98***		1.35**
		(0.57)		(0.42)
Mainly C or D, some B		-5.36***		2.59***
		(0.31)		(0.23)
Constant	46.16***	46.16***	8.82***	8.82***
	(0.26)	(0.25)	(0.19)	(0.19)
Observations	13,927	13,927	10,891	10,891
RMSE	7.74	7.63	4.74	4.69
R-squared	0.10	0.13	0.06	0.08

Note:

*** p<0.001

** p<0.01

* p<0.05.

The table illustrates the empirical detail that the collapsed two-rating classification derived in this paper offers over a one-rating classifications. We find the expected ordering of effects: Compared to A rated areas, B rated areas perform worse, followed by C rated areas, and then D rated areas. For example, children with parents at the 25th percentile of the parent income distribution growing up in only or mainly B rated tracts are expected to obtain adult household income ranks 3.4 ranks (on a scale from 1 to 100) below children growing up in only or mainly A rated tracts.

The two-rating classification breaks apart the “Only or mainly B” classification into three subclasses: “Only B”, “Mainly B, some A”, and “Mainly B, some C or D”. The best performing subclass “Mainly B, some A” is associated with a 2.2 unit reduction, while the worst performing subclass “Mainly B, some C or D” is associated with a 4.2 unit reduction in expected adult household income rank, an effect nearly twice the size. Only B rated tracts are associated with a 2.5 unit reduction, which falls in between the two aforementioned. These results are intuitive, reflecting the hazardous effects of C and D ratings and protective effects of A ratings that we observe overall, even among tracts that are predominantly covered by B rated areas.

## Discussion

This study has examined different approaches for linking past neighborhood HOLC ratings to present day census tracts to facilitate future scholarship on the long-term effects of this racist federal policy. We empirically examined different approaches for classifying census tracts in terms of historical HOLC ratings when census tracts are covered by multiple areas with different grades or only partially covered by HOLC-rated areas. We generated 54 classifications that group census tracts in different ways and seven datasets that vary thresholds for including tracts based on minimum rated HOLC area coverage. Using cross-validation with OLS regression, we identified features of classifications that improve predictive validity and identified specific classifications with high predictive validity. We derived a 10-class classification that is easy to use and interpret and achieves 90% of the predictive performance of the predictively optimal 40-class classification.

Very detailed classifications had poor predictive validity. The main exception is that if census tracts are covered by multiple ratings, classifications that consider up to two ratings (the one covering the most and the one covering the second most area) have better predictive performance than classifications considering only one rating. The 10-class classification we derive for applied research therefore includes single-letter (“Mainly A”, “Only B”, …) and two-letter classes (“Mainly B, Some A”, “Mainly D, some C”, …).

The cross-validation experiments were performed across six dependent variables including three health outcomes and three metrics of opportunity that capture observed neighborhood features (COI 2.0) and proxy-measures of unobserved features (residing in low poverty neighborhood, adult household income rank) of neighborhoods. The classification features associated with improved predictive performance varied little across dependent variables. The specific classifications identified as most predictive were among the best performing classifications for each outcome. Validation analyses illustrated that our preferred 10-class classifications identified here also performed well in predicting outcomes not used during cross-validation and performed better than similar classifications used in previous research. Altogether, these results show that the classifications identified here should have a high degree of predictive validity across many different dependent variables.

Overall, we found sizeable and statistically significant associations between historical redlining and numerous present-day census tract health and socio-economic outcomes, including a composite index of neighborhood opportunity (COI 2.0), measures of intergenerational economic mobility, poor self-rated mental and physical health, and life expectancy. These results hint at the far-reaching, cross-sectoral effects of government redlining to be unpacked further by future research.

Our results do not invalidate results from previous empirical studies. We show that previously used approaches do not perform substantially worse than the predictively optimal ones identified here. We show, however, that existing approaches could be improved in a number of ways. First, it may not be necessary to exclude tracts that are only fractionally covered by HOLC ratings or to disregard tracts that are less than 50% covered by HOLC rated areas [8, 9]. Including tracts that are only fractionally covered by HOLC-rated areas does not attenuate the relationship between HOLC grades and census tract outcomes [8]. Including tracts that are as little as 5% covered by HOLC-rated area did not degenerate predictive performance on average. These patterns are consistent with a labeling or contagion mechanism, where unrated areas in the vicinity of a rated area inherited the rating status of an area in close proximity once they undergo development. Including fractionally covered tracts increases the number of tracts in the analysis and the statistical power of tests. It also facilitates analyses for effect modification which require additional statistical power.

Second, the 10-class classification we derived explained more variation across different outcomes than simpler classifications previously used [8–10]. The classification takes seriously the fact that census tracts are often covered by differently rated HOLC polygons and leverages this information efficiently. It also provides additional margins around which to conduct causal inferences among census tracts that are identical in their primary rating, but differ in their secondary rating.

Third, our results indicate that including unrated area as a separate class is not generally warranted [10]. If an area was slated for development, it likely would have received a HOLC rating. The fact that it was unrated may reflect that the area was not developed or anticipated to be soon developed at the time, which would be consistent with treating unrated areas as missing (at random) in terms of HOLC rating status. The fact that predictive performance declines when including unrated areas is consistent with this interpretation.

Our cross-validation methodology is designed to address potential concerns that we relied on a purely empirical approach to define optimal classifications and that future applications may be vulnerable to finding associations between redlining and outcomes when there are none (Type 1 errors), because the classifications were derived by fitting many classifications to many different outcomes. Cross validation penalizes any classification or classification feature that only fits the training data well but performs poorly in the hold-out test data. Furthermore, our analysis showed that the identification of optimal classifications and classification features did not hinge on the choice of a specific dependent variable. Finally, the specific classification features and classifications identified are justified conceptually. It is reasonable to expect that secondary ratings have predictive value in addition to the primary ratings that research has considered thus far. For example, if fully B-rated census tracts are hypothesized to have better outcomes than fully C-rated areas, we would expect that a tract that is covered by both B and C ratings has outcomes that are perhaps better than fully C-rated tracts and worse than fully B-rated tracts (see Tables 4 and 6). It also makes sense that unrated areas should not be included in a HOLC rating classifications, because they were not rated by HOLC.

Our results were obtained using a dataset of all census tracts that were partially or fully covered by HOLC rated areas published by the Digital Scholarship Lab at the University of Richmond. We did not examine or quantify uncertainty related to the fact that within some subset of census tracts, e.g., for a specific city, cross-validation might identify a different classification. Or, one might prefer a different classification based on knowledge of the historic idiosyncrasies in HOLC ratings in a specific city. Because the data underlying our study will be publicly available, users can make adjustment they deem warranted, though we would caution against ad-hoc adjustments that could run the risk overfitting.

The census tract HOLC rating data we developed here answers recent calls for historically and spatially grounded metrics of structural racism [27]. HOLC neighborhood ratings are historically grounded, they provide a snapshot of a federally sanctioned racist practices in the housing sector in the 1930s that had long-term effects on racial/ethnic residential segregation and neighborhood inequities. This data allows researchers to shift the focus from present-day neighborhood conditions and present-day health outcomes to historical forces shaping both. HOLC neighborhood ratings are also explicitly spatial measures of structural racism. They capture the place-specific focus of racist policies that try to enforce spatial separation and resource inequity across White and Non-White neighborhoods. Census tract HOLC rating data therefore allows researchers to focus on the historical, racist origins of present-day neighborhood/spatial inequities. We believe that more fully incorporating the historic legacies of discrimination and racism still at play in present circumstances can only strengthen theories that attempt to explain how current circumstances, e.g. poverty, influence individual life chances.

We hope this study and its underlying data will facilitate applied research examining the impact of redlining on present day outcomes and inform community conversations on the impacts of structural racism. We believe that historical HOLC ratings provide relevant context for the development and implementation of present-day policies aiming to improve neighborhood equity. Whenever policies or programs aim to identify specific neighborhoods (census tracts) for investments, our census tract HOLC data can be used as additional data points to identify neighborhoods that were potentially impacted by racist government policies.

Redlining is one, but by no means the only example of institutional racism in the US [28–30]. More research on the long-term effects of redlining and similarly racist policies and practices in housing and other sectors is needed to unpack and redress the insidious effects of structural racism and the damage it has inflicted on non-White neighborhoods and populations. Such efforts can support public engagement processes that illuminate and policies that address structural racism in the US. We have publicly released the census tract data used to derive the classifications as well as the classifications themselves on our project website at data.diversitydatakids.org/dataset/holc_census_tracts-1930s-home-owner-loan-corporation—holc—neighborhood-grades-for-u-s—census-trac.

## Supporting information

S1 Appendix (DOCX)
