# Supplementary material for: Connecting past to present: Examining different approaches to linking historical redlining to present day health inequities
Source: PLoS One. 2022 May 19;17(5):e0267606. doi: 10.1371/journal.pone.0267606 (PMC9119533; doi:10.1371/journal.pone.0267606)
Supplement: S1 Appendix — (DOCX) [file pone.0267606.s001.docx]

**Appendix Table 1. OLS Regression coefficients from regressions of cross-validated mean squared prediction errors on experiment features, based on 2,628 cross-validation experiments.**

|  | Household income rank (p50) | Life expectancy | Low poverty neighbor-hood (p50) | Mental health | Physical health | Child Oppor-tunity Index 2.0 |
| --- | --- | --- | --- | --- | --- | --- |
| 2 ratings (Ref.) |  |  |  |  |  |  |
| 1 rating | 0.026*** | 0.027*** | 0.028*** | 0.029*** | 0.021*** | 0.043*** |
|  | (0.002) | (0.004) | (0.003) | (0.003) | (0.003) | (0.005) |
| 3 ratings | 0.006* | 0.008* | 0.006* | 0.012*** | 0.012*** | 0.006 |
|  | (0.002) | (0.004) | (0.003) | (0.003) | (0.003) | (0.005) |
| 4 ratings | 0.020*** | 0.029*** | 0.020*** | 0.034*** | 0.030*** | 0.029*** |
|  | (0.002) | (0.004) | (0.003) | (0.003) | (0.003) | (0.005) |
| 5 ratings | 0.032*** | 0.049*** | 0.034*** | 0.057*** | 0.053*** | 0.058*** |
|  | (0.003) | (0.005) | (0.003) | (0.004) | (0.003) | (0.006) |
| Rank ordered, set 6 (Ref.) |  |  |  |  |  |  |
| 50% bins, set 5 | 0.002 | 0.004 | 0.002 | 0.005 | 0.006 | 0.004 |
|  | (0.003) | (0.004) | (0.003) | (0.003) | (0.003) | (0.006) |
| 33% bins, set 4 | 0.010*** | 0.009* | 0.008** | 0.024*** | 0.025*** | 0.015** |
|  | (0.003) | (0.004) | (0.003) | (0.003) | (0.003) | (0.006) |
| 25% bins, set 3 | 0.013*** | 0.018*** | 0.018*** | 0.018*** | 0.015*** | 0.021*** |
|  | (0.003) | (0.004) | (0.003) | (0.003) | (0.003) | (0.006) |
| 20% bins, set 2 | 0.017*** | 0.019*** | 0.019*** | 0.027*** | 0.023*** | 0.024*** |
|  | (0.003) | (0.004) | (0.003) | (0.003) | (0.003) | (0.006) |
| 10% bins, set 1 | 0.042*** | 0.063*** | 0.047*** | 0.051*** | 0.048*** | 0.077*** |
|  | (0.003) | (0.004) | (0.003) | (0.003) | (0.003) | (0.006) |
| Exclude unrated (Ref.) |  |  |  |  |  |  |
| Include unrated | 0.015*** | 0.025*** | -0.001 | 0.026*** | 0.024*** | 0.027*** |
|  | (0.002) | (0.003) | (0.002) | (0.002) | (0.002) | (0.003) |
| Threshold = 5% (Ref.) |  |  |  |  |  |  |
| Threshold = 1% | -0.001 | 0.001 | -0.001 | 0.004 | -0.000 | 0.001 |
|  | (0.003) | (0.005) | (0.003) | (0.004) | (0.003) | (0.006) |
| Threshold = 10% | -0.001 | 0.001 | 0.001 | 0.002 | -0.001 | 0.001 |
|  | (0.003) | (0.005) | (0.003) | (0.004) | (0.003) | (0.006) |
| Threshold = 15% | 0.001 | 0.001 | 0.001 | 0.003 | -0.002 | 0.002 |
|  | (0.003) | (0.005) | (0.003) | (0.004) | (0.003) | (0.006) |
| Threshold = 25% | 0.002 | 0.006 | 0.000 | 0.002 | -0.002 | 0.004 |
|  | (0.003) | (0.005) | (0.003) | (0.004) | (0.003) | (0.006) |
| Threshold = 33% | 0.001 | 0.006 | 0.001 | 0.003 | -0.001 | 0.007 |
|  | (0.003) | (0.005) | (0.003) | (0.004) | (0.003) | (0.006) |
| Threshold = 50% | 0.000 | 0.006 | 0.003 | 0.003 | -0.002 | 0.008 |
|  | (0.003) | (0.005) | (0.003) | (0.004) | (0.003) | (0.006) |
| Constant | 0.840*** | 0.867*** | 0.836*** | 0.829*** | 0.869*** | 0.781*** |
|  | (0.003) | (0.005) | (0.004) | (0.004) | (0.004) | (0.007) |
| Observations | 378 | 378 | 378 | 378 | 378 | 378 |
| R-squared | 0.67 | 0.63 | 0.58 | 0.73 | 0.72 | 0.59 |

Note: *** p<0.001, ** p<0.01, * p<0.05

**Appendix Figure 1. Median MSE across experiments for each of the 54 classifications tested, pooled over all dependent variables and by dependent variable.**

Note: The yellow circle marks the best fitting classification identified in Figure 3 (three ratings, rank ordered; 40 levels). The blue circle is the most parsimonious classification that is closest to the minimum averaged MSE identified in Figure 3 (two ratings, rank ordered; 16 levels). The top row labeled “Pooled” reports classification-specific median MSEs computed over experiments for all outcomes. The remaining rows report classification-specific median MSEs computed over experiments for a given outcome.

**Appendix Table 2. RMSE statistics from OLS regressions of 11 census tract level health and socio-economic outcomes on different census tract HOLC rating measures/classifications.**

|  | Proportions | One rating | Rank-ordered,  2 ratings, collapsed | Rank-ordered,  2 ratings, detailed | Rank-ordered,  3 ratings, optimal |
| --- | --- | --- | --- | --- | --- |
| **Degrees of Freedom** | 3 | 3 | 9 | 15 | 39 |
|  |  |  |  |  |  |
| *Health Outcomes* |  |  |  |  |  |
| Drinking | 1.130 | 1.131 | 1.124 | 1.123 | 1.121 |
| Cancer | 1.001 | 1.010 | 0.999 | 0.999 | 0.998 |
| Asthma | 0.994 | 0.999 | 0.990 | 0.990 | 0.989 |
| CHD | 1.167 | 1.167 | 1.157 | 1.157 | 1.156 |
| Smoking | 0.995 | 1.000 | 0.984 | 0.983 | 0.981 |
| Diabetes | 1.098 | 1.102 | 1.092 | 1.092 | 1.090 |
| Phys. act. | 0.987 | 0.995 | 0.983 | 0.983 | 0.980 |
| Obesity | 1.045 | 1.048 | 1.032 | 1.032 | 1.028 |
|  |  |  |  |  |  |
| *Average* | *1.052* | *1.057* | *1.045* | *1.045* | *1.043* |
|  |  |  |  |  |  |
| *Economic outcomes* |  |  |  |  |  |
| Income rank | 0.982 | 0.987 | 0.973 | 0.972 | 0.969 |
| Income in top 20% | 0.959 | 0.964 | 0.954 | 0.953 | 0.950 |
| Low pov. neighborhood | 0.912 | 0.921 | 0.909 | 0.909 | 0.906 |
|  |  |  |  |  |  |
| *Average* | *0.951* | *0.958* | *0.946* | *0.944* | *0.941* |

Note: All outcome were standardized using the z-score transformation.
